# Age‐ and Sex‐Specific Optimal Cutoffs for Obesity Indices for Early Detection of Metabolic Syndrome Risk in Taiwanese Adults

**DOI:** 10.1111/cob.70117

**Published:** 2026-10-27

**Authors:** Chien‐Yeh Hsu, Jia‐Wen Guo

**Affiliations:** ^1^ Department of Information Management National Taipei University of Nursing and Health Sciences Taipei Taiwan; ^2^ Master Program in Global Health and Health Security, College of Public Health Taipei Medical University Taipei Taiwan; ^3^ College of Nursing University of Utah Salt Lake City Utah USA

**Keywords:** body mass index, Chinese visceral adiposity index, triglyceride glucose index, visceral fat

## Abstract

Age‐ and sex‐specific obesity index cutoffs may improve the early identification of individuals at risk for metabolic syndrome. This study determined optimal cutoff points for body mass index (BMI), triglyceride glucose index (TyGI) and Chinese visceral adiposity index (CVAI) to predict MetS risk in Taiwanese adults. This retrospective study using the Mei Jau Health Database, spanning 2008 to 2013, included 30 382 adults (76% females) aged 18 years or older. Subjects free of metabolic syndrome in 2008 had additional data recorded from 2009 to 2013. Approximately 8.5% developed MetS after baseline (2008). Significant differences in BMI, TyGI and CVAI between sexes (*p* < 0.001) were observed, with females exhibiting lower values than males. The optimal cutoff values for obesity‐related indices were determined, stratified by age groups (≤ 29, 30–39, 40–49, ≥ 50) and sex. BMI cutoffs: 22.14–23.14 (females), 23.54–24.94 (males); TyG: 4.44–4.63 (females), 4.55–4.69 (males); CVAI: 10.70–77.11 (females), 67.02–82.23 (males). Most of these age‐ and sex‐specific cutoff values exhibited AUCs and sensitivity values greater than 0.70 and demonstrated acceptable to excellent predictive performance. In summary, CVAI cutoffs showed greater accuracy and sensitivity than BMI and TyGI for identifying MetS risk in Taiwanese adults without baseline MetS.

## 1 Introduction

Metabolic syndrome (MetS) [1, 2] presents a significant global health concern, particularly among Asian populations, including Taiwan [2]. The prevalence of MetS in Taiwan has increased from 9.5% to 25.5% in earlier reports (1993–2001) [3, 4] to 34.6% in the recent Nutrition and Health Survey in Taiwan (NAHSIT) 2017–2020 [5]. MetS risk rises with age, influenced by sex‐related hormonal differences [5, 6, 7, 8]. For example, males tend to accumulate visceral fat, increasing dyslipidaemia, hypertension and insulin resistance [9], while premenopausal women have better insulin sensitivity, which declines after menopause, increasing abdominal fat and dyslipidaemia [10]; consequently, women over 50 show a higher incidence of MetS than men [6, 11].

Obesity, particularly visceral obesity, is a key driver of MetS. Accurate, accessible measures of obesity are crucial for predicting its onset. Body mass index (BMI) [12, 13], triglyceride glucose index (TyGI) [14, 15, 16] and Chinese visceral adiposity index (CVAI) [17] are obesity‐related indices useful for identifying MetS risk in Chinese populations, including Taiwanese. Higher values of these indices correlate with greater MetS risk. BMI is a popular screening method to determine obesity, providing a straightforward assessment of body fat using height and weight, and it is known to be affected by factors such as age, sex and race [18]. For example, Asians often have lower BMI levels compared to Caucasians [18, 19], possibly due to ethnicity‐specific differences; therefore, the World Health Organization (WHO) suggested an Asia‐Pacific BMI classification: underweight (below 18.5 kg/m^2^), normal (18.5–22.9 kg/m^2^), overweight (23.0–24.9 kg/m^2^) and obesity (over 25.0 kg/m^2^) [13]. While BMI does not distinguish between fat and muscle mass [18], other obesity‐related indices, such as the TyGI and CVAI, were developed to assess visceral fat and also MetS risk. The TyGI contributes insights into lipid metabolism and glucose homeostasis by combining measurements of fasting triglyceride and glucose levels [15, 20]; elevated TyGI values indicate disruptions in lipid metabolism and impaired glucose regulation, highlighting its role in identifying metabolic disturbances [20]. CVAI is calculated by integrating age, anthropometric parameters (BMI and waist circumference) and biochemical markers (triglycerides and HDL‐C) into sex‐specific formulas tailored for the Chinese population to assess visceral obesity [17]. Its value lies in its precise measurement of visceral adiposity among Chinese populations and in identifying metabolic health risks.

The development of MetS is influenced by sex‐ and age‐specific patterns, as well as lifestyle factors [6, 21, 22], which differ by sex and may be differentially associated with MetS; for example, alcohol consumption, smoking and physical activity have been more strongly associated with MetS in males [21, 22]. Older women aged 50 or 60 and above exhibit a higher incidence of MetS than men [6, 11]. Additionally, as people age, their susceptibility to obesity increases, thereby heightening the risk of MetS [6, 11, 23]. Therefore, it is essential to adopt a sex‐ and age‐specific approach to improve current screening processes for identifying individuals at high risk of MetS. To achieve more precise evaluations of MetS risk across diverse populations, determining BMI, TyGI and CVAI cutoff values should meticulously consider age and sex factors [14]. This study aims to establish clinically feasible cutoff values for three obesity‐related indices—BMI, TyGI and CVAI—to improve MetS risk prediction in Taiwanese adults, stratified by age and sex, using data from individuals without a prior MetS diagnosis.

## 2 Methods

### 2.1 Study Sample

A retrospective study used data from the Mei Jau (MJ) Health Management Institution, a private health screening service in Taiwan. All participants provided written informed consent before each health check‐up. We requested adults aged ≥ 18 years who underwent health assessments between 2008 and 2013 from the MJ database and only included eligible participants who had: (1) baseline assessments in 2008 and at least one follow‐up between 2009 and 2013; (2) no MetS at baseline and (3) complete data on demographics, metabolic indicators and lifestyle factors (e.g., exercise type, drinking status and smoking status). Of the 34 541 participants in the requested dataset, 4159 (12%) were excluded due to missing data, leaving 30 382 eligible participants; all included participants were free of MetS at baseline.

### 2.2 Definition of MetS

MetS was defined using modified National Cholesterol Education Program (NCEP) criteria for Asians [24]: (1) fasting glucose ≥ 100 mg/dL, (2) SBP ≥ 130 mmHg or DBP ≥ 85 mmHg, (3) triglycerides ≥ 150 mg/dL, (4) low high‐density lipoprotein cholesterol (HDL‐C) < 50 mg/dL in female or < 40 mg/dL in men and (5) waist ≥ 80 cm in female or ≥ 90 cm in men. Individuals meeting three or more criteria between 2009 and 2013 were classified as MetS (+) in this study.

### 2.3 Definition and Calculations of BMI, TyGI and CVAI

BMI was calculated using the following formula:

$$\mathrm{BMI}=\text{weight}\;\left(\mathrm{kg}\right)/\text{height}\;{\left(m\right)}^{2}$$

Asians typically have lower BMI levels than Caucasians; therefore, the World Health Organization (WHO) suggested an Asia‐Pacific BMI classification [13], which was different from the original WHO BMI classification: underweight (below 18.5 kg/m^2^), normal (18.5–22.9 kg/m^2^), overweight (23.0–24.9 kg/m^2^) and obesity (over 25.0 kg/m^2^).

TyGI was calculated using the following formula [16], which was corrected from the original publication [15]:

TyGI=Ln[fasting triglyceride(mg/dL)×fasting glucose(mg/dL)]/2

Higher TyGI values indicate poorer insulin sensitivity and an increased risk of MetS, while lower values suggest the opposite [16]; a TyGI above 4.7 [25], calculated using the corrected version of the original formula [15], is considered as a cutoff value for impaired fasting glucose and metabolic diseases.

CVAI were calculated using the following equations [17, 18]:

$$\begin{array}{c} \text{CVAI for female}=-187.32+1.71\times \mathrm{Age}\;\left(\text{year}\right)+4.23\times \mathrm{BMI}\;\left(\mathrm{kg}/m^{2}\right)\\ \;+1.12\times \text{waist circumference}\;\left(\mathrm{cm}\right)+39.76\\ \;\times \mathrm{Log}10\;\text{triglyceride}\;\left(\text{mmol}/L\right)-11.66\\ \;\times \mathrm{HDL}-C\;\left(\text{mmol}/L\right) \end{array}$$

$$\begin{array}{c} \text{CVAI for male}=-267.93+0.68\times \mathrm{Age}\;\left(\text{year}\right)+0.03\times \mathrm{BMI}\;\left(\mathrm{kg}/m^{2}\right)\\ \;+4.00\times \text{waist circumference}\;\left(\mathrm{cm}\right)\\ \;+22\times \mathrm{Log}10\;\text{triglyceride}\;\left(\text{mmol}/L\right)\\ \;-16.32\times \mathrm{HDL}-C\;\left(\text{mmol}/L\right) \end{array}$$

Higher CVAI score indicates a greater level of visceral fat, which is associated with an increased risk of MetS, cardiovascular and cerebrovascular diseases [17, 26], while a lower CVAI suggests less visceral fat and a reduced risk.

### 2.4 Data Analysis

Data were analysed by sex, age groups and MetS development status. Independent‐sample *t*‐tests were conducted for continuous variables and *χ* ^2^ tests for categorical variables to assess differences by sex and MetS development status across demographic characteristics, metabolic indicators and lifestyle factors. Age was categorized into < 30, 30–39, 40–49 and ≥ 50 years to reflect clinically meaningful stages of metabolic risk progression, as individuals in their 30s often exhibit early metabolic changes, those in their 40s show a higher prevalence of metabolic abnormalities, and those aged ≥ 50 years experience further risk increases associated with ageing and hormonal changes, thereby improving the clinical relevance and readability of the results.

Primary logistic regression models were fitted to evaluate the associations of BMI, TyGI and CVAI with MetS. Sensitivity analyses were then conducted by additionally adjusting for lifestyle variables (drinking, smoking and exercise) in corresponding logistic regression models. The Akaike Information Criterion (AIC) was used to evaluate and compare model fit between the sensitivity and primary models to assess robustness.

To identify optimal cutoff values of BMI, TyGI and CVAI for predicting MetS risk by sex and age group, the dataset was randomly split into training and testing sets at a ratio of 70% and 30%, respectively. In the training set, optimal cutoff values were estimated using receiver operating characteristic (ROC) analysis with the *cutpointr* R package, selecting points that maximized the product of sensitivity and specificity. The identified cutoffs were subsequently evaluated in the test set to assess their performance [27].

## 3 Results

### 3.1 Characteristics of the Sample

Of 30 382 eligible subjects, 76% were female, with a mean age of 40.9 ± 11 years (Table 1). Although females constituted the majority of the sample, this study conducted sex‐specific analyses to minimize potential bias and ensure independent evaluation of each sex. Females generally had lower BMI, TyGI and CVAI values than males. Females had a higher proportion of normal BMI, lower risk of developing MetS, drank and smoked less and engaged in less intense exercise than males, highlighting sex differences in lifestyle (Table 2). Overall, 8.5% (*n* = 2584) later developed MetS, with males generally younger than females, highlighting sex‐specific differences in age of onset. As expected, those who later developed MetS had higher BMI, TyGI and CVAI, were more often overweight, drank and smoked more and engaged in less intense exercise (Table 2). Notably, the lifestyle variables were highly skewed in the cohort (~90% drinking < 1 time/week, ~90% no cigarette use and ~80% low‐intensity exercise), indicating limited variability and reduced suitability for threshold‐based analyses, such as cutpoint determination. However, they could still be used in sensitivity analysis to evaluate potential residual confounding and the impact of adjustment on the associations with MetS.

**TABLE 1:** Demographic and lifestyle characteristics by sex.

Variables	Female	Male	Total	*χ* ^2^	*p*
	(*n* = 23 057, 75.9%)	(*n* = 7325, 24.1%)	(*N* = 30 382)		
	*n* (%)	*n* (%)	*n* (%)		
Age				637.67	< 0.001
≤ 29	2760 (12.0)	1379 (18.8)	4139 (13.6)		
30–39	8184 (35.5)	3088 (42.2)	11 272 (37.1)		
40–49	6763 (29.3)	2015 (27.5)	8778 (28.9)		
≥ 50	5350 (23.2)	843 (11.5)	6193 (20.4)		
BMI classification^a^				2431.91	< 0.001
Underweight	2728 (11.8)	216 (2.9)	2944 (9.7)		
Normal	14 345 (62.2)	3023 (41.3)	17 368 (57.2)		
Overweight	3471 (15.1)	2091 (28.5)	5562 (18.3)		
Obesity	2513 (10.9)	1995 (27.2)	4508 (14.8)		
Drinking					
< 1 time/week	21 836 (94.7)	5841 (79.7)	27 677 (91.1)	1535.41	< 0.001
1–2 times/week	855 (3.7)	1058 (14.4)	1913 (6.3)		
≥ 3 times/week	366 (1.6)	426 (5.8)	792 (2.6)		
Exercise				3458.74	< 0.001
Low‐intensity	20 262 (87.9)	4239 (57.9)	24 501 (80.6)		
Moderate‐intensity	2444 (10.6)	2260 (30.9)	4704 (15.5)		
High‐intensity	351 (1.5)	826 (11.3)	1177 (3.9)		
Smoking				3027.03	< 0.001
0 cigarette/day	22 039 (95.6)	5476 (74.8)	27 515 (90.6)		
< 10 cigarettes/day	649 (2.8)	717 (9.8)	1366 (4.5)		
≥ 10 cigarettes/day	369 (1.6)	1132 (15.5)	1501 (4.9)		

^a^ BMI categories were defined according to the WHO Asia‐Pacific criteria as: BMI < 18.5 kg/m^2^ (underweight), 18.5–22.9 kg/m^2^ (normal weight), 23.0–24.9 kg/m^2^ (overweight) and ≥ 25.0 kg/m^2^ (obesity) [13].

**TABLE 2:** Metabolic syndrome‐related variables by sex.

Variables	Female	Male	Total	*χ* ^2^	*p*
	(*n* = 23 057, 75.9%)	(*n* = 7325, 24.1%)	(*N* = 30 382)		
	*n* (%)	*n* (%)	*n* (%)		
MetS development status^a^				110.37	< 0.001
MetS (+)	1742 (7.6)	842 (11.5)	2584 (8.5)		
MetS (−)	21 315 (92.4)	6483 (88.5)	27 798 (91.5)		

	*M* (SD, range)	*M* (SD, range)	*M* (SD, range)	*t*	*p*
Age (years)	41.8 (11.2, 18–92)	37.9 (9.8, 18–84)	40.9 (11.0, 18–92)	29.0	< 0.001
Fasting glucose (mg/dL)	95.4 (11.6, 60–386)	98.3 (8.4, 70–275)	96.1 (11.0, 60–386)	−23.81	< 0.001
Triglyceride (mg/dL)	79 (42.1, 11–1919)	108 (58.6, 12–751)	86.1 (48.3, 11–1919)	−39.80	< 0.001
HDL‐C (mg/dL)	67.1 (15.0, 25–189)	54.5 (11.6, 27–171)	64 (15.2, 25–189)	74.96	< 0.001
LDL‐C (mg/dL)	109 (29.8, 16–377)	118 (29.4, 24–305)	111 (30.0, 16–377)	−23.52	< 0.001
Waist circumference (cm)	70.2 (6.5, 52–116)	80.4 (7.21, 57–140)	72.6 (8.0, 52–140)	−109.0	< 0.001
SBP (mmHg)	110 (15.0, 71–200)	117 (12.2, 64–200)	112 (14.7, 64–200)	−38.4	< 0.001
DBP (mmHg)	65.9 (9.8, 40–129)	71.2 (9.2, 44–130)	67.3 (9.8, 40–130)	−40.1	< 0.001
BMI (kg/m^2^)	21.5 (2.9, 13.9–44.6)	23.5 (2.8, 14.4–43.7)	22 (3.0, 13.9–44.6)	−52.9	< 0.001
TyGI	4.4 (0.2, 3.4–6.1)	4.6 (0.3, 3.6–5.6)	4.5 (0.2, 3.4–6.1)	−50.6	< 0.001
CVAI	29.8 (35, −64.4–210.2)	58.1 (34.2, −56.2–297.2)	36.6 (36.9, −64.4–297.2)	−61.4	< 0.001

Abbreviations: BMI, body mass index; CVAI, Chinese visceral adiposity index; DBP, diastolic blood pressure; HDL‐C, high‐density lipoprotein cholesterol; LDL‐C, low‐density lipoprotein cholesterol; MetS, metabolic syndrome; SBP, systolic blood pressure; TyGI, triglyceride‐glucose index.

^a^ MetS development status: MetS (+) for participants who developed metabolic syndrome (2009–2013) and MetS (−) for those who did not.

### 3.2 Sensitivity Analyses: Primary vs Lifestyle‐Adjusted Models of BMI, TyGI and CVAI in Relation to MetS

In the primary analyses, logistic regression models were used to evaluate the associations of BMI, TyGI and CVAI with MetS. All three indices were significantly associated with MetS in both female and male groups.

In the primary analyses, logistic regression models were used to evaluate the associations of BMI, TyGI and CVAI for MetS. All three indices were significantly associated with MetS in both female and male groups (all *p* < 0.001; Table 3). Among the three models, CVAI demonstrated the best model fit, as indicated by the lowest AICs in both groups. In sensitivity analyses, additional lifestyle variables were included and coded as binary variables because of limited variation and skewed distributions. After further adjustment, the associations of BMI, TyGI and CVAI with MetS remained virtually unchanged, supporting the robustness of the primary findings. Although the sensitivity models showed slightly improved model fit after including lifestyle variables, the estimated associations between BMI, TyGI, CVAI and MetS remained largely unchanged (Table 3).

**TABLE 3:** Primary and sensitivity analyses of BMI, TyGI and CVAI in relation to metabolic syndrome.

Primary predictor	Primary analysis			Sensitivity analysis		
	OR (95% CI)	*p*	AIC	OR (95% CI)	*p*	AIC
Female (*n* = 23 057)						
BMI	1.430 (1.406, 1.455)	< 0.001	10331.9	1.428 (1.405, 1.453)	< 0.001	10306.9
TyGI	1.586 (1.551, 1.623)	< 0.001	10452.5	1.583 (1.548–1.619)	< 0.001	10433.2
CVAI	1.047 (1.045, 1.049)	< 0.001	8929.2	1.047 (1.045, 1.049)	< 0.001	8924.4
Male (*n* = 7325)						
BMI	1.344 (1.308, 1.381)	< 0.001	4713.7	1.345 (1.309, 1.382)	< 0.001	4673.8
TyGI	1.414 (1.370, 1.459)	< 0.001	4711.9	1.403 (1.359, 1.449)	< 0.001	4708.2
CVAI	1.035 (1.032, 1.038)	< 0.001	4432.1	1.035 (1.032, 1.037)	< 0.001	4426.1

*Note:* Primary logistic regression models evaluated primary BMI, TyGI and CVAI separately as predictors of MetS. Sensitivity models additionally adjusted for lifestyle variables (drinking, exercise and smoking), coded as binary variables: drinking (< 1 time/week vs. ≥ 1 time/week), exercise (low intensity vs. moderate/high intensity) and smoking (0 cigarettes/day vs. > 0 cigarettes/day). ORs for TyGI are presented per 0.1‐unit increase. For reference, the corresponding ORs per 1‐unit increase were 100.932 (95% CI, 80.684–126.608) and 98.868 (95% CI, 79.008–124.058) in females for the primary and sensitivity analyses, respectively, and 31.879 (95% CI, 23.301–43.821) and 29.486 (95% CI, 21.467–40.689) in males, respectively. ORs for BMI and CVAI are expressed per 1‐unit increase.

Abbreviations: AIC, Akaike information criterion; BMI, body mass index; CI, confidence interval; CVAI, Chinese visceral adiposity index; OR, odds ratio; TyGI, triglyceride–glucose index.

### 3.3 Optimal Cutoff Values for Baseline BMI, TyGI and CVAI

The optimal BMI cutoff values for the female age groups ranged from 22.14 to 23.14, categorizing individuals as normal (18.5–22.9 kg/m^2^) or overweight (23.0–24.9 kg/m^2^), based on the WHO Asian BMI classification [13]. For men, all optimal BMI cutoff values ranging from 23.54 to 24.94 among the age groups fall within the overweight category. Lower cutoff values of BMI were observed in females compared to males across each age group. The TyGI cutoff values were similar between females and males in each age group, with values in female age groups (4.44–4.63) being slightly lower than those in male age groups (4.55–4.69). The CVAI cutoff values varied widely between female age groups (10.70–77.11) and male age groups (67.02–82.23); the cutoff values increased with age in both sexes (Table 4).

**TABLE 4:** Optimal cutoff values for BMI, TyGI and CVAI in the various age groups and sex to predict the development of metabolic syndrome using baseline data.

	Age group	Cutoff >=	AUC (95% CI)^a^	Specificity	Sensitivity
Female (*n* = 23 057)					
BMI^b^	≤ 29	22.14	0.90 (0.83, 0.98)	0.80	0.82
	30–39	22.27	0.85 (0.81, 0.88)	0.77	0.75
	40–49	22.74	0.82 (0.78, 0.85)	0.69	0.77
	≥ 50	23.14	0.74 (0.71, 0.77)	0.64	0.69
	All	22.40	0.82 (0.80, 0.84)	0.77	0.72
TyGI	≤ 29	4.44	0.86 (0.77, 0.95)	0.80	0.79
	30–39	4.44	0.83 (0.79, 0.87)	0.85	0.68
	40–49	4.48	0.79 (0.75, 0.83)	0.78	0.64
	≥ 50	4.63	0.70 (0.66, 0.73)	0.55	0.75
	All	4.48	0.79 (0.78, 0.81)	0.79	0.67
CVAI	≤ 29	10.70	0.96 (0.93, 0.98)	0.93	0.86
	30–39	33.44	0.90 (0.87, 0.93)	0.82	0.83
	40–49	49.38	0.87 (0.84, 0.90)	0.82	0.80
	≥ 50	77.11	0.79 (0.77, 0.82)	0.73	0.74
	All	50.28	0.88 (0.86, 0.89)	0.84	0.77
Male (*n* = 7325)					
BMI^b^	≤ 29	24.94	0.86 (0.79, 0.93)	0.84	0.79
	30–39	24.37	0.71 (0.65, 0.77)	0.66	0.66
	40–49	24.45	0.74 (0.68, 0.79)	0.63	0.70
	≥ 50	23.54	0.64 (0.54, 0.73)	0.65	0.55
	All	24.32	0.73 (0.69, 0.76)	0.67	0.67
TyGI	≤ 29	4.55	0.70 (0.61, 0.80)	0.56	0.69
	30–39	4.63	0.72 (0.67, 0.76)	0.67	0.64
	40–49	4.69	0.73 (0.68, 0.78)	0.67	0.66
	≥ 50	4.68	0.63 (0.54, 0.73)	0.54	0.63
	All	4.63	0.72 (0.69, 0.75)	0.67	0.65
CVAI	≤ 29	67.02	0.86 (0.80, 0.91)	0.64	0.81
	30–39	70.58	0.76 (0.71, 0.81)	0.64	0.69
	40–49	78.22	0.80 (0.76, 0.85)	0.77	0.72
	≥ 50	82.23	0.72 (0.64, 0.80)	0.81	0.54
	All	70.58	0.79 (0.76, 0.81)	0.74	0.67

*Note:* Cutoff values were derived from the training set, whereas AUC, sensitivity and specificity were evaluated in the test set.

Abbreviations: AUC, area under the receiver operating characteristic curve; BMI, body mass index; CVAI, Chinese visceral adiposity index; TyGI, triglyceride‐glucose index.

^a^ AUC values range from 0.5 (no discrimination) to 1.0 (perfect discrimination) and were interpreted as follows: ≥ 0.90, outstanding; 0.80–0.89, excellent; 0.70–0.79, acceptable and 0.51–0.69, poor [27].

^b^ BMI categories were defined according to the WHO Asia‐Pacific criteria as: BMI < 18.5 kg/m^2^ (underweight), 18.5–22.9 kg/m^2^ (normal weight), 23.0–24.9 kg/m^2^ (overweight) and ≥ 25.0 kg/m^2^ (obesity) [13].

When evaluating the performance of each optimal cutoff value across female and male age groups, similar results were observed in the training and testing sets, indicating consistent performance. Table 4 presents the results from the testing set to evaluate the derived cutoff values. Most AUCs for BMI, TyGI and CVAI demonstrated excellent to acceptable discriminatory power, with values of 0.70 or higher. These AUCs effectively distinguished between individuals who developed MetS and those who did not across most age groups in both sexes. Additionally, CVAI consistently demonstrated higher AUCs and sensitivity values compared to the BMI and TyGI cutoff values across all age groups in both females and males (Table 4). For the two AUCs < 0.70 observed in male subgroups aged ≥ 50 years for BMI (AUC 0.64) and TyGI (AUC 0.63), these estimates should be interpreted with caution due to smaller sample sizes compared with other subgroups, which may lead to less stable estimates; therefore, these findings should be interpreted cautiously. In summary, these findings suggest that CVAI may be a more reliable indicator for identifying the risk of MetS in the Taiwanese population, in both females and males.

## 4 Discussion

This study established the cutoff predictive values of BMI, TyGI and CVAI for MetS risk in a Taiwanese population, stratified by age groups for both females and men. First, we identified significant associations between obesity‐related indices and MetS. Second, among the age‐ and sex‐specific cutoff values established, CVAI exhibited the optimal performance to identify MetS risk. Notably, females generally presented with lower BMI, TyGI and CVAI values than men, emphasizing the necessity of sex‐specific considerations in metabolic health assessment and intervention. Additionally, older individuals of both sexes were more likely to exhibit higher BMI, TyGI and CVAI cutoff values, indicating age‐related variations in these indices. Therefore, this study highlights the need for age‐ and sex‐specific diagnostic criteria in predicting MetS risk.

In this study, the cumulative incidence of MetS from 2009–2013 was 8%. This measure is not directly comparable to prevalence estimates reported in previous studies. Prior analyses using MJ health screening data reported prevalence estimates of 9.5% (8% in females and 11% in males) in 2000–2001 [4] and 12.7% (8% in females and 18% in males) in 1998–2002 [11], whereas national data from NAHSIT 2017–2020 reported a substantially higher prevalence of 34.6% (30.0% in females and 39.3% in males) [5]. These differences likely reflect variation in study populations. The MJ database represents a fee‐based private health check‐up cohort, whereas NAHSIT is nationally representative. Accordingly, the MJ cohort may capture metabolic risk patterns within a health‐screening population, potentially with higher socioeconomic status; therefore, the derived obesity‐related index cutoffs should be interpreted with caution when applied to populations with different socioeconomic characteristics, where MetS risk may be higher, particularly among lower socioeconomic groups.

A higher proportion of female participants (76%) was observed in the MJ database, which may limit the precision of estimates in the male subgroup. This imbalance likely reflects real‐world participation patterns in preventive health screening, where women are more likely to undergo health examinations. However, the male sample size remained substantial (*n* = 7325), providing adequate statistical power for subgroup analyses. As this was a secondary analysis of the MJ Health database, the sex distribution could not be modified and likely reflects real‐world differences in participation in health screening, where women are more likely to undergo health examinations [28, 29].

A consistent finding in this study, as well as in previous research, is that males exhibit a higher prevalence of MetS and tend to develop it at an earlier age than females [4, 11, 23, 30]. The observed sex‐ and age‐specific differences in MetS are likely influenced by a combination of biological, behavioural and lifestyle factors, including hormonal changes, variations in fat distribution, dietary habits and physical activity levels [7, 8]. These findings highlight the importance of incorporating sex‐ and age‐specific considerations into MetS screening and prevention to improve early detection and optimize care across different demographic groups.

Lifestyle factors (drinking, smoking and exercise) are well established to play an important role in the development of MetS [21, 22]. However, in the present study, sensitivity analyses showed no significant influence of these factors on the associations of BMI, TyGI and CVAI with MetS. This finding was not unexpected, as the analyses were based on baseline data when all participants were free of MetS. In addition, these lifestyle variables were self‐reported, subject to recall bias, lacked sufficient detail (e.g., drinking frequency without quantity and broadly defined exercise intensity) and were highly skewed, with most participants reporting low alcohol consumption, non‐smoking and low‐intensity exercise. Potentially, they may not fully capture true behavioural exposures.

BMI has traditionally been used to identify individuals at risk of developing MetS, but a well‐documented limitation of BMI is its inability to accurately differentiate between visceral fat and overall body mass, particularly among individuals with obesity [1]. This limitation arises from BMI's calculation, which is based solely on body weight relative to height, without accounting for variations in fat distribution, muscle mass or differences due to age and sex. As a result, individuals with a BMI above 25.0 kg/m^2^ may not necessarily have excessive visceral fat, while those with a lower BMI could still be at risk due to fat accumulation in metabolically active regions. In this study, the age‐ and sex‐specific BMI cutoff values identified predominantly fell within the normal or overweight range according to standard BMI classification. These findings suggest that individuals who do not meet the conventional BMI criteria for obesity may still be at risk for MetS, even in the absence of apparent MetS manifestations.

Given the limitations of BMI in accurately reflecting visceral fat, incorporating additional indices such as TyGI and CVAI, which better capture metabolic dysfunction and visceral adiposity, may provide a more precise assessment of MetS risk. TyGI has been shown to be significantly associated with MetS in Taiwanese, Chinese and Korean populations [14, 20, 30, 31, 32, 33]. Our findings are consistent with these previous studies. CVAI provides a more comprehensive assessment of MetS risk by incorporating age, waist circumference, triglycerides, lipid profiles and BMI, unlike BMI, which primarily reflects overall body weight, and TyGI, which mainly focuses on triglyceride and glucose levels.

Since CVAI was originally developed for the Chinese population [17] and has consistently demonstrated strong predictive performance for MetS risk in previous studies [32, 34], it was not surprising that the CVAI cutoff values identified in this study outperformed those of BMI and TyGI in predicting MetS risk among both females and males. This superior performance is likely attributable to the inclusion of multiple MetS‐related components in the calculation of CVAI, such as BMI, waist circumference, triglycerides and HDL‐C [17, 18], which may provide an advantage over simpler indices in identifying individuals at higher risk for MetS. Therefore, these findings suggest that CVAI may serve as a more effective screening tool for the Taiwanese population.

Noticeably, this study identifies lower cutoff values for BMI, the TyGI and CVAI compared to previous research [11, 14, 31, 32, 33, 34]. The TyGI cutoff values identified in this study (4.44–4.63 for females; 4.55–4.69 for males) were lower than those reported in previous studies among Chinese adults (8.7–8.9) [31, 32, 34] and Korean adults (8.8–8.9) [14, 33]. This discrepancy may arise from the use of the original TyGI formula published in 2008 [15] in these earlier studies, rather than the corrected version introduced by the authors in 2020 [16]. Moreover, the TyGI cutoff values identified across female and male age groups in this study were consistently lower than the widely recognized threshold of 4.7 [25]. Regarding the CVAI cutoff values identified in this study (10.70–77.11 for female; 67.02–82.23 for men), they were also significantly lower than those reported in previous studies among Chinese adults (105.3–114.4 for female; 111.5–119.1 for men) [32, 34]. This discrepancy may be explained by differences in study design. Previous studies primarily compared obesity‐related indices between participants with and without MetS, whereas our study focused on early detection by deriving cutoff values from baseline data of individuals without MetS manifestations, including those who later developed MetS within 5 years. Consequently, lower cutoff values for obesity‐related indices were identified in this study, which may be more suitable for identifying individuals at high risk of developing MetS before clinical onset.

Adopting age‐ and sex‐specific cutoff values is crucial for improving the accuracy of MetS risk assessment. Most cutoffs in this study demonstrated acceptable to outstanding performance based on AUCs. Although CVAI showed the best predictive performance for identifying individuals at higher risk of MetS, it requires more variables than BMI and TyGI, including laboratory measures, which may limit its feasibility in primary care settings. In contrast, simpler indices such as BMI and TyGI may be more practical when data are limited. Therefore, in practice, the choice of obesity‐related index may depend on the availability of clinical and laboratory data, and users should consider whether the improved predictive performance of CVAI justifies its additional data requirements compared with simpler indices such as BMI or TyGI.

### 4.1 Strengths and Limitations

This study has several notable strengths. First, only participants without MetS at baseline were included and followed longitudinally to identify incident MetS, allowing clear temporal sequencing and strengthening causal interpretation compared with cross‐sectional approaches. Second, the study used a large, community‐based sample, enhancing the generalizability of the findings, particularly among individuals who regularly participate in health check‐ups. Third, this study identified optimal cutoffs with good AUCs for three widely used obesity‐related indices—BMI, TyGI and CVAI—supporting their use in early screening of MetS risk. Despite these strengths, several limitations of this study should be acknowledged. The study relied on data from the MJ health screening database [4], which may introduce selection bias due to the associated health screening fee. This financial barrier could limit the generalizability of our findings to populations with different socioeconomic backgrounds, particularly those with limited access to preventive healthcare services. Additionally, the absence of comprehensive medical records restricted our ability to account for other potential contributing factors, such as genetic predisposition, medication use and comorbid conditions, which may influence MetS risk. Moreover, information on family history and dietary factors was not available in the database for this study; therefore, we were unable to account for these factors in the analysis, which may lead to incomplete adjustment and influence the study results. Nevertheless, this study provides valuable insights into the early detection of MetS among previously undiagnosed individuals. By leveraging a large population sample and a robust analytical approach, our findings contribute to a better understanding of how obesity‐related indices can be utilized in clinical practice for risk assessment and preventive intervention. Finally, external validation using independent or nationally representative databases (e.g., NAHSIT or the Taiwan Biobank) was not performed in this study because access to these databases was unavailable; therefore, the generalizability of these findings requires further confirmation. Finally, the large ORs observed for TyGI when expressed per 1‐unit increase (see footnote in Table 3) should be interpreted cautiously. Large effect estimates in retrospective analyses may be susceptible to estimation instability under certain data conditions [35]. In the present study, the large sample size, 8.5% MetS incidence and consistent sensitivity‐analysis estimates make sparse‐data instability less likely. To improve interpretability, TyGI associations are reported per 0.1‐unit increase in Table 3, although these ORs should still be interpreted as measures of association rather than direct estimates of risk.

## 5 Conclusion

This study established age‐ and sex‐specific cutoff values for BMI, TyGI and CVAI to improve the precision of metabolic health assessment in the Taiwanese population. Applying these tailored cutoff values may facilitate personalized screening and earlier identification of individuals at risk for MetS. Future studies should conduct external validation using independent or nationally representative cohorts and further evaluate the clinical utility of these cutoff values in healthcare practice.

## Author Contributions

Study concept and design: C.‐Y.H. and J.‐W.G. Acquisition of data: C.‐Y.H. Data analysis and management: J.‐W.G. Interpretation of data analysis: C.‐Y.H. and J.‐W.G. Statistical analysis: J.‐W.G. Drafting of the manuscript: J.‐W.G. Critical revision of the manuscript: C.‐Y.H. and J.‐W.G. Study supervision: C.‐Y.H.

## Funding

The authors have nothing to report.

## Ethics Statement

All eligible subjects provided their consent by signing a written informed consent form prior to each health screening visit. The study protocol was approved by the Research Ethics Committee of National Taiwan University (Protocol/Approval Number: 202412EM051).

## Conflicts of Interest

The authors declare no conflicts of interest.

## Data Availability

The data that support the findings of this study are available from Mei Jau (MJ) Health Management Institution. Restrictions apply to the availability of these data, which were used under license for this study. Data are available from the author(s) with the permission of Mei Jau (MJ) Health Management Institution.
